# An Integrative Approach for Mapping Differentially Expressed Genes and Network Components Using Novel Parameters to Elucidate Key Regulatory Genes in Colorectal Cancer

**DOI:** 10.1371/journal.pone.0133901

**Published:** 2015-07-29

**Authors:** Manika Sehgal, Rajinder Gupta, Ahmed Moussa, Tiratha Raj Singh

**Affiliations:** 1 Department of Biotechnology and Bioinformatics, Jaypee University of Information Technology (JUIT), Waknaghat, Solan, H.P. 173234, India; 2 LabTIC Laboratory, ENSA, Abdelmalek Essaadi University, Tangier, Morocco; University of Georgia, UNITED STATES

## Abstract

For examining the intricate biological processes concerned with colorectal cancer (CRC), a systems biology approach integrating several biological components and other influencing factors is essential to understand. We performed a comprehensive system level analysis for CRC which assisted in unravelling crucial network components and many regulatory elements through a coordinated view. Using this integrative approach, the perceptive of complexity hidden in a biological phenomenon is extensively simplified. The microarray analyses facilitated differential expression of 631 significant genes employed in the progression of disease and supplied interesting associated up and down regulated genes like *jun*, *fos* and *mapk1*. The transcriptional regulation of these genes was deliberated widely by examining transcription factors such as *hnf4*, *nr2f1*, *znf219* and *dr1* which directly influence the expression. Further, interactions of these genes/proteins were evaluated and crucial network motifs were detected to associate with the pathophysiology of CRC. The available standard statistical parameters such as *z*-score, *p*-value and significance profile were explored for the identification of key signatures from CRC pathway whereas a few novel parameters representing over-represented structures were also designed in the study. The applied approach revealed 5 key genes i.e. *kras*, *araf*, *pik3r5*, *ralgds* and *akt3* via our novel designed parameters illustrating high statistical significance. These novel parameters can assist in scrutinizing candidate markers for diseases having known biological pathways. Further, investigating and targeting these proposed genes for experimental validations, instead being spellbound by the complicated pathway will certainly endow valuable insight in a well-timed systematic understanding of CRC.

## Introduction

Colorectal cancer (CRC) influences millions of people worldwide and exists as the most commonly diagnosed cancers after lung and breast cancer [1]. CRC contributes to second largest cause of death in males and third highest in females, also prevalence of the disorder is observed mostly in the economically developed regions [2, 3]probably due to lifestyle and dietary issues. The incidence and mortality rate for CRC is approximately 35–40 percent higher in men as compared to women [4]. As per the cancer status in United States for 2013, approximately 102,480 peoplesuffered and 50,830 died of CRC which governs the severity of disease [5]. CRC mainly manifests as abnormal growth of cells occurring at the lining of colon or rectum and the disease progression takes place by replacing a non-cancerous polyp to cancerous tumour. Previous reports [6–8] suggest a variety of factors linked to the disease pattern such as inflammatory bowel disease, polyps, obesity, smoking and genetic history of cancer. The disease is also characterized by rectal bleeding, obstruction, abdominal pain, lack of appetite and subsequent weight loss [7, 9]. None of the symptoms independently assures the incidence of CRC and often there are no observable symptoms in early CRC. Therefore, appropriate screening for the disease is required [10] to facilitate early detection and timely removal of polyps [11].

In order to identify biomarkers for early detection, the cancer pathway and disease progression has to be critically examined. Although, in recent decades, many studies have conceded on screening, diagnosis and treatment for CRC [12, 13] but still the genetic and initiation factors accountable for the disease are unknown [14]. There is a huge lack in understanding of mechanisms underlying the progression of CRC from non-cancerous polyp to a tumor and their responsible pathways [15]. Studies illustrate that CRC is mainly associated with chromosome instability (CIN) [16] and microsatellite instability (MSI) pathways [17, 18].Genetic aberrations in genes involved in CIN pathway leads to the activation of oncogenes like *kras* and inactivate certain tumor suppressor genes such as *smad4*, *p53*, *smad2*, *bax* and *apc* [19]. Moreover, previous reports [20] and a database on DNA repair genetic association studies [21] suggests that mutations in DNA repair genes, i.e. *mlh1*, *msh2*, *msh3* and *msh6*of MSI pathway contributes to hereditary non-polyposis colorectal cancer (HNPCC) and CRC. Therefore, investigating important up and down regulated genes may deduce markers for CRC as observed in other studies for different diseases [22]. Further, a comprehensive perceptive on the genes and related pathways is required for designing specific and effective therapies for CRC [23].

There is already a massive accumulation of gene expression data for CRC in public domains and several computational techniques have been applied for its analysis. But, the ultimate challenge lies in extracting vital biological information or markers from this amalgamation of data [24]. The DNA microarray technique not only provides a valuable measure for estimating expression of thousand genes at once but also offers vital molecular clues regarding mechanisms underlying the pathophysiology of disease [22, 25]. Subsequently, the strategy we pursued includes identification of biologically significant genes and elucidation of key patterns or motifs formed by these candidate genes which governs the functional impact of various biological processes in CRC. Each identified gene was then annotated focussing on the categorization of genes by means of biological processes, molecular functions and cellular components for their association and involvement in CRC [26].

Additionally, an attempt was made to identify vital network components (network motifs) occurring in elevated frequencies than randomly expected in a pathway. These network motifs provide statistically overrepresented sub-structures (sub-graphs) in a network and are recognized as simple building blocks of a complicated network. These network motifs play a central role in recognition and analysis of specific patterns in biological networks and yield significant insights in better understanding of complex biological processes involved in intricate human diseases [27]. We applied computational and statistical criterion for the efficient detection of biological network motifs in CRC and their functional evaluation measures were utilized to reduce the complexity for recognizing best appropriate candidates in the proposed study.

The main perspective of our study was system-component analyses for CRC with several biological components comprising the expression of genes involved, their annotations, and analyses in form of complex network motifs governing vital functions. The foremost objective was to manually curate and annotate all genes, network components, processes, molecular functions and pathways involved in CRC and then facilitate identification of a few key genes that may serve as vital markers for CRC. On the whole, an integrative approach was practised that includes various aspects of molecular data, biomarkers, networks and pathways for uncovering the intricacy in CRC pathway and then confining the search to only a few genes or network components that may answer diverse biological queries concerning CRC. Also, such *in silico* approach could be applied to other diseases in quest for identifying biomarkers and the study will not only assist experimental biologists, geneticists and other scientific community to identify novel biomarkers for diseases but also has implications for the pharmaceutical industry to target important molecules and design appropriate target-based drugs for medications.

## Materials and Methods

**Fig 1:**
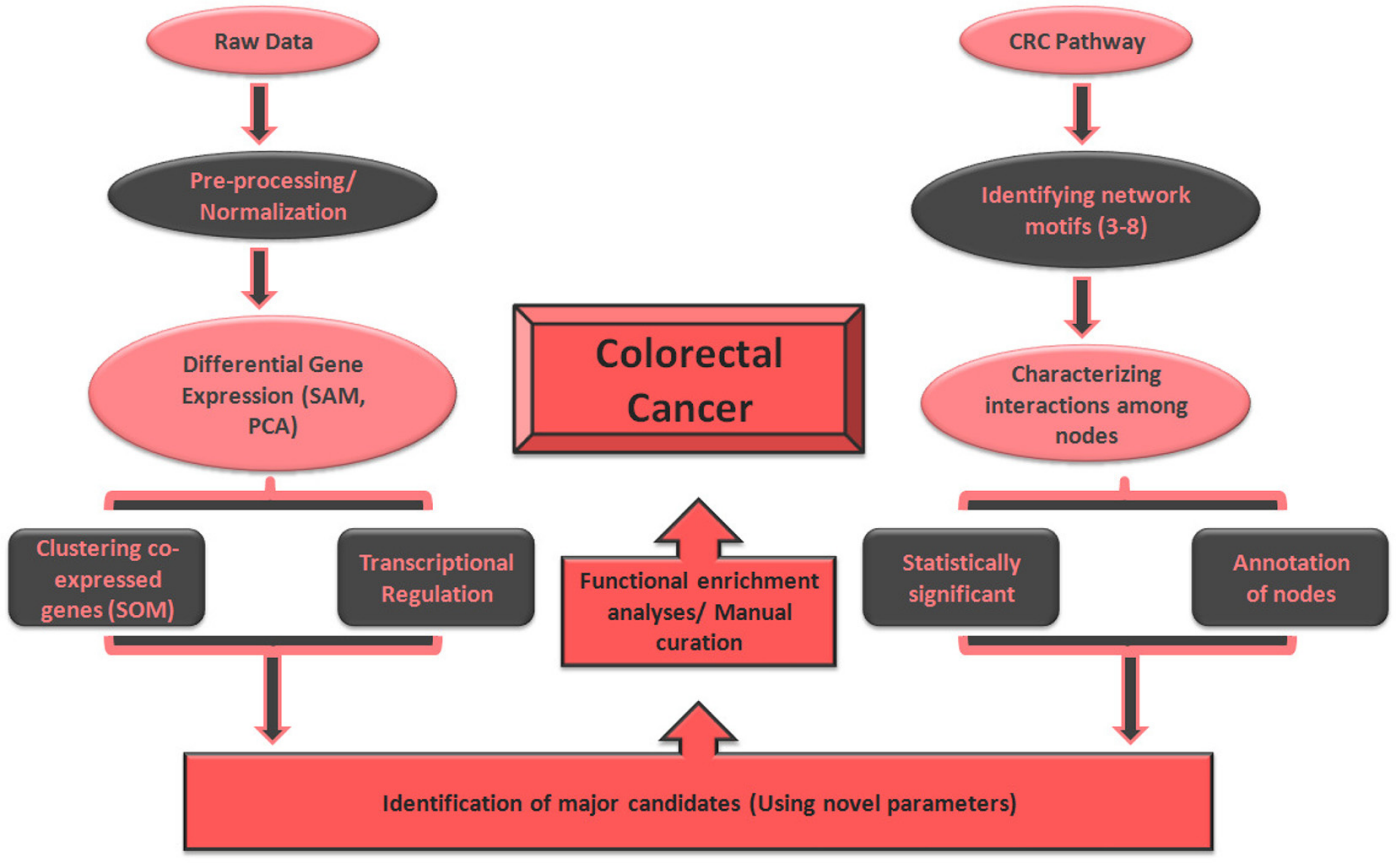
The methodology applied for recognizing biomarkers in colorectal cancer. Study initiated with the characterization of differentially expressed genes in colorectal cancer dataset and their transcriptional regulation. Important interactions and network patterns were identified from the CRC pathway and eventually functional enrichment was executed for key players in the disease progression.

An *in silico* approach with different forms of raw data, computational tools, software and databases was applied for extensive understanding of mechanisms involved in CRC. A myriad of in-house perl scripts and statistical techniques were employed for characterization of biomarkers for the disease. Entire workflow representing different parameters and biological aspects considered for the study is presented in Fig 1.

### Biological data

The DNA microarray analysis was performed on raw data retrieved from Gene Expression Omnibus (GEO) [28] for the early onset of CRC [29]. The main priority for studying gene expression at an early stage was to identify biomarkers for early detection of disease which consequently could then be aptly managed. The ultimate goal of the study was to detect additional differentially expressed genes in early onset CRC since the one’s involved in familial adenomatous polyposis (FAP) [30] and HNPCC [31, 32] are already well illustrated. The extracted dataset was then analyzed using GeneChip U133-Plus 2.0 Array. Furthermore, the network motifs for CRC were detected by retrieving biological pathways from KEGG [33], Reactome [34], BioGRID [35] and other pathway databases [36].

### Pre-processing of data

First and the foremost step for DNA microarray analysis is pre-processing and normalization of raw data which then is subjected to further analysis. This process minimizes the noise resulting from technical variations and subsequently permits data to be compared for determining the actual biological changes. The implementation of data normalization assists in stabilizing unequal quantities of starting RNA, differences in labelling or detection efficiencies between the used fluorescent dyes and systematic biases in expression levels. Hence, the data congregated from each available CRC disease chip has been normalized using the robust multi average analysis (RMA) algorithm [37] from Microarray Data Analysis System (MIDAS) in TM4 microarray software suite.

### Identification of differentially expressed genes

Subsequent to microarray experiments, recognizing genes with altered expression profiles in diseased state is an imperative and tedious task to perform. The multiple hypotheses testing problem is generally observed due to the presence of a few conditions, many observations and thousands of hypotheses to be explicitly tested. To overcome this issue, an appropriate statistic has been chosen for testing each gene in the dataset and then computing its corresponding *p*-value. An adjustment process is applied to the raw *p*-values in order to avoid errors from hypotheses multiplicity [38] and finally a QQ plot is generated. This plot represents the values of observed test statistics against the expected test statistics under a combination of null hypotheses. Ultimately, the expressed genes for control and diseased states were considered for significance analysis of microarrays (SAM) and volcano plot analyses to measure the substantial gap leading to the identification of crucial regulatory genes [39, 40].

### Cluster analysis for co-expressed genes

The clustering of differentially expressed genes was characterized using hierarchical clustering algorithm. Genes sharing similar expression profiles and other biological features were clustered together and vice-versa. In earlier studies, this kind of classification is achieved for diverse forms of cancers but for CRC, a poor classification has been observed [41]. Moreover, hierarchical clustering was performed to deduce the significance of differential expression selection step in classifying the co-regulated genes. Further, for the identification of important patterns and components in multi-dimensional microarray data, principal component analysis (PCA) was accomplished [42]. This technique facilitated the detection of major principal components and aided in analyzing and visualizing genes with similar expression profiles.

### Transcriptional regulation of CRC genes

Since, gene regulation plays crucial role at the level of transcription by employing a variety of transcription factors (TFs) and their target genes; a broad knowledge of transcriptional regulatory elements (REs) is necessary for thorough understanding of gene regulation and underlying complex regulatory processes. Available, *in silico* tools such as DiRE (Distant Regulatory Elements) [43] and oPOSSUM [44] were surveyed for the identification of REs among these differentially expressed genes. Both the toolsassist in identification of TFs where DiRE has a unique feature of recognizing REs outside of proximal promoter regions by considering full gene locus. The REs including proximal promoters and distant REs like enhancers, repressors and silencers were detected for a broader perspective on the concerned regulatory process of CRC.

### Functional enrichment for differentially expressed genes

The enrichment analysis focused on manual curation and annotation via WEB-based Gene SeT AnaLysis Toolkit (WebGestalt) [45] and Gorilla tools. The former tool comprises of genomics, proteomics and large-scale genetic studies generated data for functional annotation of differentially expressed and co-expressed datasets. This toolkit integrates information from several public resources and often provides accurate and sensitive results, aiding in identification of biological processes, their cellular compartments and molecular functions associated with the corresponding genes. Whereas, GOrilla tool [46] makes computation on the basis of exact *p*-values without simulation analyses for detecting the functional characteristics of the gene sets. Both the tools make use of same statistical approach i.e. hyper-geometric distribution (HGD) for significance testing and functional enrichment of genes whereas WebGestalt furthermore exploits Fisher’s exact test for the annotation analyses. Mathematically, for HGD if there are *‘N’* number of genes in a group where *‘A’* genes are related to a particular GO term and a sample of *‘n’* genes from *‘N’* is taken, then the probability of acquiring *‘a’* genes associated with *‘a’* or more GO terms in a sample *‘n’* is deliberated using HGD:

$$p-value=1-\sum_{i=0}^{a-1}f_{HG}\left(i;N,A,n\right)=1-\sum_{i=0}^{a-1}\frac{\left(\begin{array}{c} A\\ i \end{array}\right)\;\left(\begin{array}{c} N-A\\ n-i \end{array}\right)}{\left(\begin{array}{c} N\\ n \end{array}\right)}$$

GOrilla displays the statistically significant and enriched genes at the top of ranked gene list and uses a variant of regular HGD named mHG (minimum hypergeometric) for the enrichment analyses of ranked gene lists [47]. In many cases, a fixed threshold *(n)* doesn’t work and ranking of all the elements (genes) is required for finding the value of *‘n’* that further minimizes HGD. For instance, consider a ranked gene list say *g*_*1*_,…, *g*_*N*_ in place of a target set, and defined label vector: *λ = λ*_*1*_,…,*λ*_*N*_ *∈ {0*, *1}* ^*N*^ as indicated by the association of ranked genes to a given GO term, *λ*_*i*_ *= 1* if *g*_*i*_ is associated with the term [47]. Then, mHG score is given by:

$$mHG\left(\lambda \right)=\;\underset{1\leq n\leq N}{min}\left(HGT(N,K,n,k_{n}\left(\lambda \right))\right)$$

Where

$$k_{n}\left(\lambda \right)=\sum_{i=1}^{n}\lambda_{i}$$

Here, the cut-off between top rated genes and rest of the genes is calibrated in a precise manner to maximize the gene enrichment analyses.

### Detection of crucial patterns from CRC pathway

Examination of vital network motifs, an important aspect to recognize the modularity and to solve large-scale structure of complicated biological networks was facilitated from complex CRC disease pathway. A variety of motif detection tools like MFinder [48], MAVisto [49] and FANMOD [50] were employed to identify motifs; where all these tools implement different algorithms. MFinder uses a semi-dynamic programming algorithm in order to reduce the run time in detecting network motifs and performs full enumeration of the sub-graphs whereas MAVisto tool employs a flexible algorithm for the identification of network motifs and also includes an advanced force-directed layout algorithm [51] for its analyses. Moreover, FANMOD runs a much sophisticated algorithm named RAND-ESU [52] that works on both directed as well as undirected networks for specification and sampling of sub-graphs. This algorithm performs better than its counter algorithms [48] for the identification of network motifs from complex biological networks.

The statistical implication of these generated motifs was then evaluated using available standard constraints such as *z*-scores, *p*-values and significance profile (SP). The *p*-value and *z*-score for each motif was estimated (via Fanmod’s output) and those having *z*-score>2 and *p*-value<0.05 were classified as significant motifs and are demonstrated in S1 Table. Further, the SP furnishes normalized *z*-score values for a particular network motif *(m*_*i*_ *)* which is given by:

$$SP\left(m_{i}\right)=\frac{Z\left(m_{i}\right)}{\sqrt{\sum_{i=1}^{n}Z{\left(m_{i}\right)}^{2}}}$$

Where *Z(m*_*i*_ *)* corresponds to the *z*-score value for each network motif.

All the generated 4–8 node sub-graphs with unique network motif IDs were then extensively analysed for examining the genes and their complex interactions in CRC using our novel designed parameters such as *‘FN*_*i*_ *’*, *‘FTN*_*i*_ *’* and *‘FT*_*i*_ *’* as represented in Table 1. The Network Motif Image ID column presents the network motif IDs as the adjacency matrix created for each interaction where 0 and 1 correspond to no connection and connection among nodes respectively.

**Table 1:** Values of the designed parameters for each particular network motif in order to deduce crucial network components.

Network Motif Image ID	Abbreviations	*FTN*_*i*_	*FN*_*i*_	*FT*_*i*_
'0000001000011000'	4a	76	25	0.329
'0000000000011100'	4b	48	16	0.333
'0000000000001110'	4c	16	16	1
'0000010000010000000101000'	5a	30	8	0.267
'0000000000000010000111000'	5b	15	6	0.4
'0000000000000100100010100'	5c	60	14	0.233
'000000000000000010000001000001110000'	6a	36	8	0.222
'000000000000000000000010001000110100'	6b	36	14	0.389
'000000000000010000000010100000001100'	6c	60	12	0.2
'000000000000000100000010000001110000'	6d	36	14	0.389
'000000000000000000000010011000100100'	6e	36	8	0.222
'000000100000000010000001010000010000'	6f	18	8	0.444
'000000100000010000000001010000000010'	6g	18	8	0.444
'000000000000000000000001000001111000'	6h	6	6	1
'0000000000000000000000000100000001000100001101000'	7a	49	18	0.367
'0000000000000000000000000000000001000110001100100'	7b	21	8	0.381
'0000000000000000010000000010000000100000011100000'	7c	21	8	0.381
'0000000000000000001000000010000000100000011100000'	7d	21	8	0.381
'0000000100000000000100000001010000001000000000100'	7e	21	9	0.429
'0000000100000001000000000010010000000000010000100'	7f	21	9	0.429
'0000000000000000000000000000000000100011001110000'	7g	14	8	0.571
'0000000000000000000000000001000001000010001110000'	7h	14	14	1
'0000000000000000000000000001000000100010001110000'	7i	14	8	0.571
'0000000000000000001000000010100000001000000011000'	7j	49	12	0.245
'0000000000000000010000000100010000000001001010000'	7k	42	10	0.238
'0000000000000000000000010000000001011000000001100'	7l	42	10	0.238
'0000000000000000001000100000000001010000000011000'	7m	56	13	0.232
'0000000000000000000000010000000001001000001001100'	**7n**	**70**	**12**	**0.171**
'0000000000000000010000000100000001001000001010000'	7o	50	13	0.26
'0000000000000000000000000000001000000010000100000110000010000100'	8a	48	10	0.208
'0000000000000000000000000000010000000010001000000100000010011000'	8b	56	12	0.214
'0000000000000000000000000000001010000000000010000110000000010100'	8c	48	12	0.25
'0000000000000000000000000000000000010000000000100110000010001100'	8d	48	10	0.208
'0000000000000000000000000010000000000010000010001100000000010100'	8e	48	11	0.229
'0000000000000000000000000000001000000001000100000110000010000100'	8f	48	10	0.208
'0000000000000000000100000000100001000000000000100000000110100000'	8g	48	11	0.229
'0000000000000000000000000000010000100000000000100100000010011000'	8h	64	13	0.203
'0000000000000000000100000000100000000100000000100100000010100000'	8i	48	11	0.229
'0000000000000000000100000000100001000000000000100000100010100000'	8j	48	11	0.229
'0000000000000000000000000000001000100000010000001000000000011100'	8k	40	12	0.3
'0000000000000000000000000010000000000010100000000100010000011000'	8l	32	11	0.344
'0000000000000000000000000000100000000100000000100010000011010000'	8m	29	13	0.448
'0000000000000000000000000000100000000100001000000000000111010000'	8n	24	10	0.417
'0000000000000000000010000000001000000100000000010000000111000000'	8o	24	9	0.375
'0000000010000000010000000000010001000000000000100000000100001000'	8p	24	10	0.417
'0000000000000000000010000000001000010000000000010000000111000000'	8q	24	9	0.375
'0000000000000000000010000000001000000100000100000000000111000000'	8r	24	9	0.375
'0000000000000000000000000000100000000100001000000000010011010000'	8s	24	10	0.417
'0000000010000000000010000000000101000000010000000000010000000010'	8t	24	10	0.417
'0000000000000000000100000000100000000100010000000100000010100000'	8u	16	9	0.563
'0000000000000000000100000100000000000001010000000000100010100000'	8v	16	9	0.563
'0000000000000000000000000000000000000001000100000000110011100000'	8w	16	10	0.625
'0000000000000000000000000000000000000000000000100011100011000100'	8x	8	8	1
'0000000000000000000000000000010000000010000000010000000111100000'	8y	8	8	1
'0000000000000000000000000000100000000010000000010000000111100000'	8z	8	8	1

Here, *‘FN*_*i*_ *’* corresponds to the number of genes present in a given network motif ID; *‘FTN*_*i*_ *’* is the sum of frequencies for all the genes occurring in a given network motif ID and *‘FT*_*i*_ *’* is defined as the ratio of number of genes for a particular network motif ID and the sum of frequencies for all genes in a given network motif. For a given network motif ID say *‘n*_*i*_ *’*, where i = 1,2,3,….,n; *‘FT*_*i*_ *’* is given by:

$$FT_{i}=\frac{FN_{i}}{FTN_{i}}$$

Each *‘FT*_*i*_ *’* value for a particular network motif ID provides the magnitude of all genes involved in a particular network motif. Thus, the applied methodology comprises of both top-down and bottom-up approaches for detecting the key players in CRC pathway. Using the top-down approach, first the entire CRC pathway was partitioned into smaller sub-graphs with small functional modules and then the involved nodes were identified and annotated. On the other hand, a bottom-up approach was applied for classifying the interactions and relationships among the nodes. Ultimately, outcome from both the approaches was incorporated to identify key nodes in CRC pathway in order to deduce the crucial genes employed in disease.

## Results

**Fig 2:**
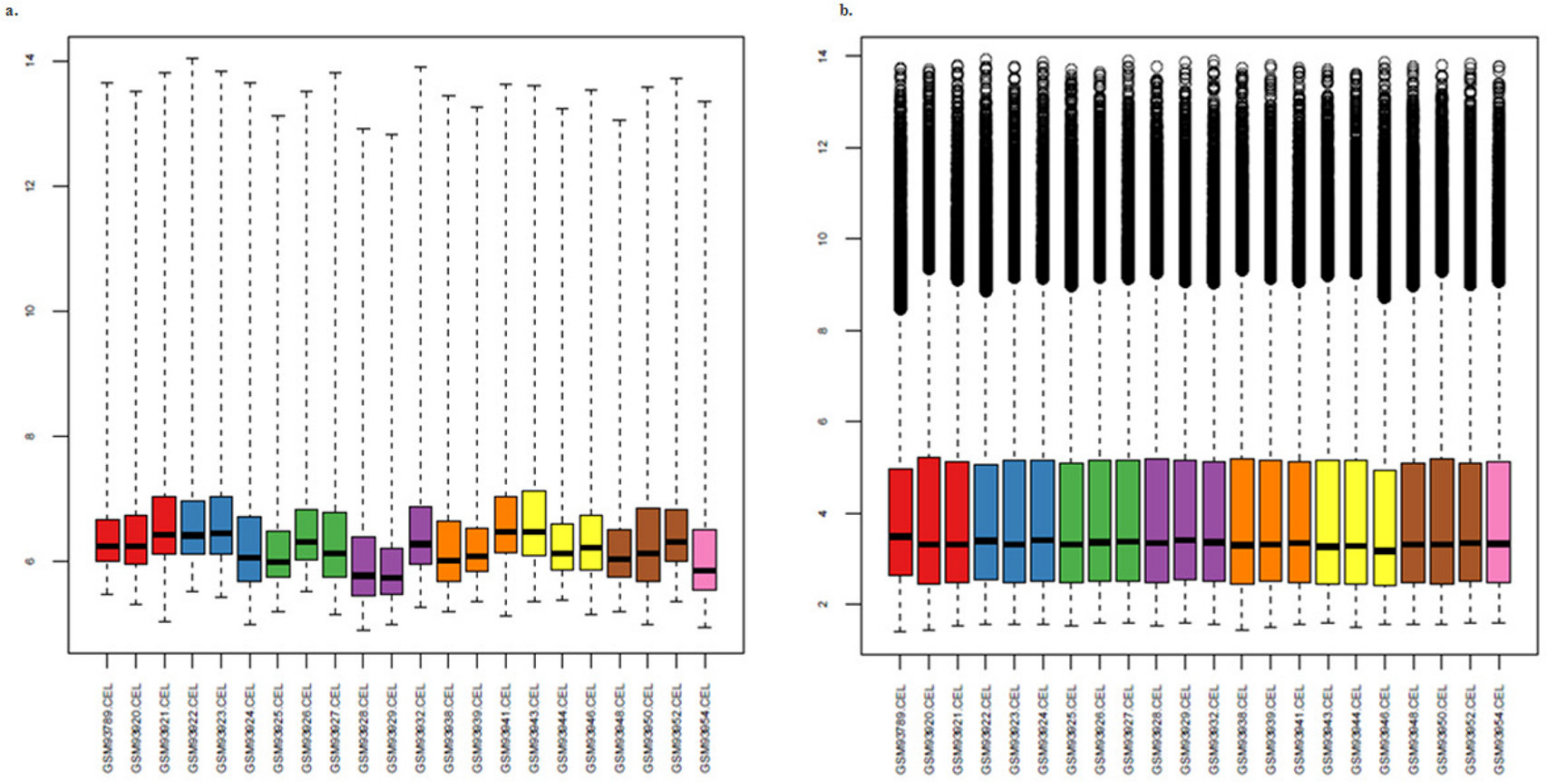
Pre-processing and normalization of DNA microarray data. 2a shows the distribution of microarray files before normalization and 2b explains the uniform distribution obtained after implementing normalization i.e. removal of noise from data.

In this study, a comprehensive analysis for differentially expressed genes, TFs, interacting proteins, putative network motifs and their implications in diverse pathways related to CRC has been extensively carried out. Selected CRC dataset for DNA microarray was considered for the process of normalization for removal of errors and noise from the dataset as depicted in Fig 2. The figure illustrates the box plot for all four Affymetrix chips before and after normalization using quantile normalization and clearly demonstrates the impact of normalization step by rectifying the signal of genes across all chips.

**Fig 3:**
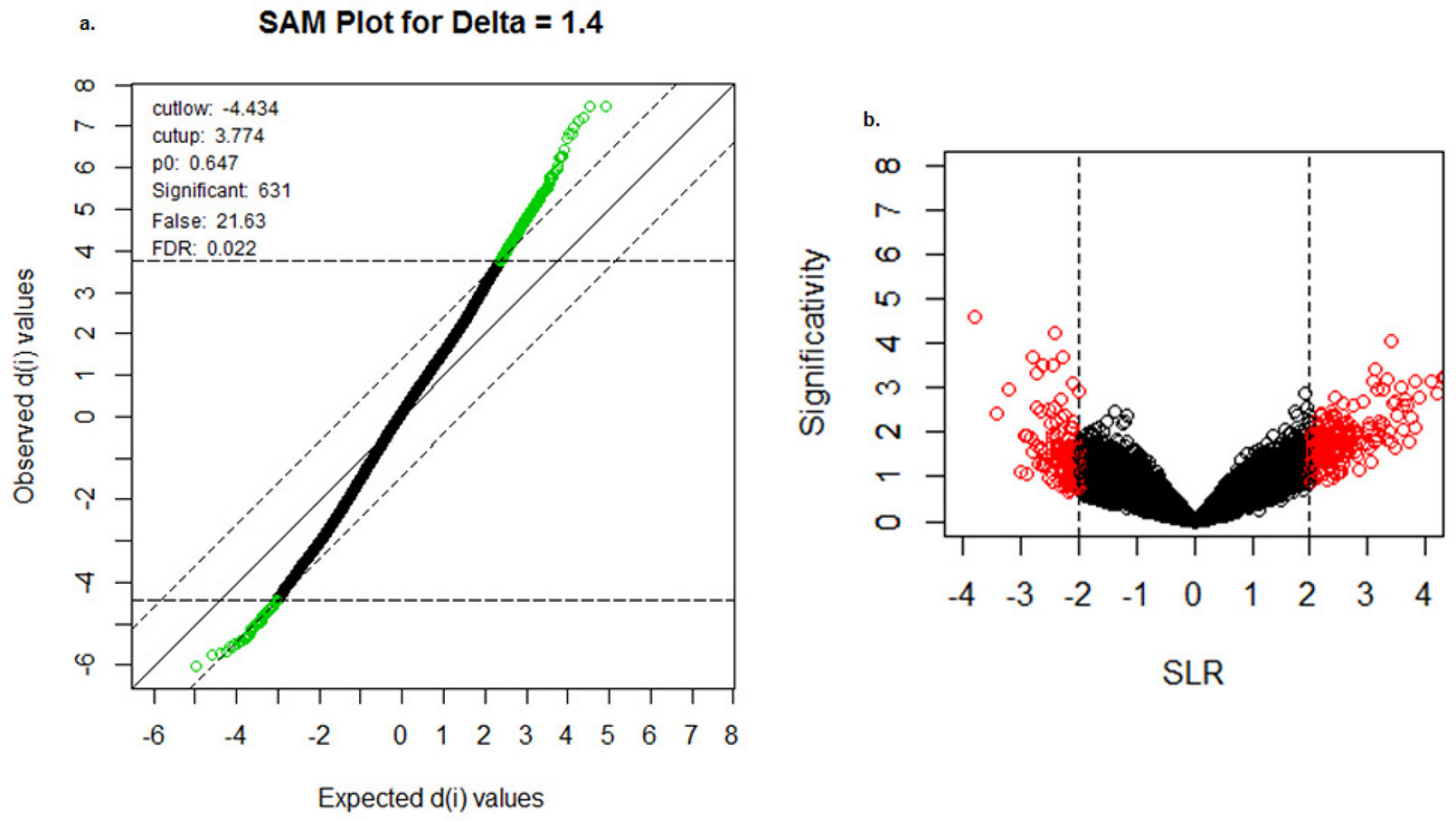
Identification of differential expression. Significance analysis of microarrays (SAM) and volcano plot were generated for detecting the differentially expressed genes in the early colorectal cancer dataset. In SAM, 631 significant genes were identified for their over or under expression in the diseased state whereas the volcano plot evidently elucidates the differentially expressed genes with red spots having signal log ratio (SLR)>2 or SLR<2.

The microarray dataset was examined for the identification of specific patterns or markers that may differentiate normal vs. diseased state for signifying the susceptibility and facilitate early diagnosis of CRC. After preliminary pre-processing and manual inspection based on the proportional analysis, final set subjected to SAM composed of only the robust candidates (see S2 Table). SAM revealed a total of 631 genes (Fig 3A) from the microarray dataset which were differentially expressed among the tested conditions since data points lie aside the diagonal line in a substantial manner. The volcano plot between control and the diseased state for CRC clearly elucidated the difference between genes that were differentially expressed in the two groups as shown in Fig 3B. Here, the spots represented in black are the genes showing normal expression whereas the red ones with signal log ratio (SLR)>2 are over expressed and those with SLR<-2 are under expressed genes in the diseased state. Moreover, SOM significant clusters are depicted in S1 Fig and PCA (well described in S2 and S3 Figs) revealed the projections for 3 different conditions, i.e. over-expressed genes, under-expressed genes and genes showing normal expression.

After characterizing the differential expression pattern of crucial genes implicated in early CRC progression, role of RE and transcriptional regulation was essential to recognize. We identified a total of 108 TFs in the gene expression dataset for CRC (S3 Table), represented in descending order of their occurrence in the frequency column. Additionally, importance of these TFs were estimated using an optimization procedure that considers a weight *‘w*_*i*_ *’* for each *i* ^*th*^TF, as a measure of its association with the input gene set and further calculates the importance value as the product of TF occurrence (frequency) and TF weight. We also classified TFs (see S4 Table) found in each differentially expressed gene from CRC dataset, providing total number of TFs for each gene, locus, their names, position and their associated types. Moreover, families for all the important TFs have been recognized and illustrated in S5 Table. We also compiled a list for top 10 TFs implicated in genes responsible for differential expression in early CRC with their frequencies of occurrence, importance and other essential details as depicted in Table 2. A few experimental validations complementing to the association of these transcription factors in CRC are also referred in the table.

**Table 2:** Identified major transcription factors in early colorectal cancer progression.

**Transcription Factor**	Frequency	Importance	JASPAR ID_1_	Class	Family	Pubmed IDs/ Experimental Databases_2_
**HNF4**	31.80%	0.31802	MA0114.1	Zinc-coordinating	Hormone-nuclear Receptor	19048623, 22731903, 22308320
**NR2F1**	19.43%	0.50044	MA0017.1	Zinc-coordinating	Hormone-nuclear Receptor	The Human Protein Atlas
**DR1**	17.31%	0.04112	-	Zinc-coordinating	Hormone-nuclear Receptor	The Human Protein Atlas, 10690519, 19251712
**PPARG**	14.49%	0.03622	MA0066.1	Zinc-coordinating	Hormone-nuclear Receptor	19186181, 16489531
**HNF1**	14.13%	0.36064	MA0046.1, MA0153.1	Helix-Turn-Helix	Homeo	12730871, 20096102
**HNF4_DR1**	13.78%	0.16882	-	Zinc-coordinating	Hormone-nuclear Receptor	22383578, 18180275
**PPAR_DR1**	13.43%	0.13428	-	Zinc-coordinating	Hormone-nuclear Receptor	11840453,25961905
**HNF4ALPHA**	12.01%	0.29848	MA0114.1	Zinc-coordinating	Hormone-nuclear Receptor	25961905, The Human Protein Atlas, 22731903
**PAX4**	12.01%	0.18322	MA0068.1	Helix-Turn-Helix	Homeo	12970747, The Human Protein Atlas, 19395656
**ER**	10.60%	0.08216	MA0112.2, MA0258.1	Zinc-coordinating	Hormone-nuclear Receptor	20663982

^1^The JASPAR IDs correspond to the transcription factors from JASPAR database

^2^The Pubmed IDs/ Experimental Databases column contains the information for literature references and databases created on experimentally validated data for their association with colorectal cancer.

**Fig 4:**
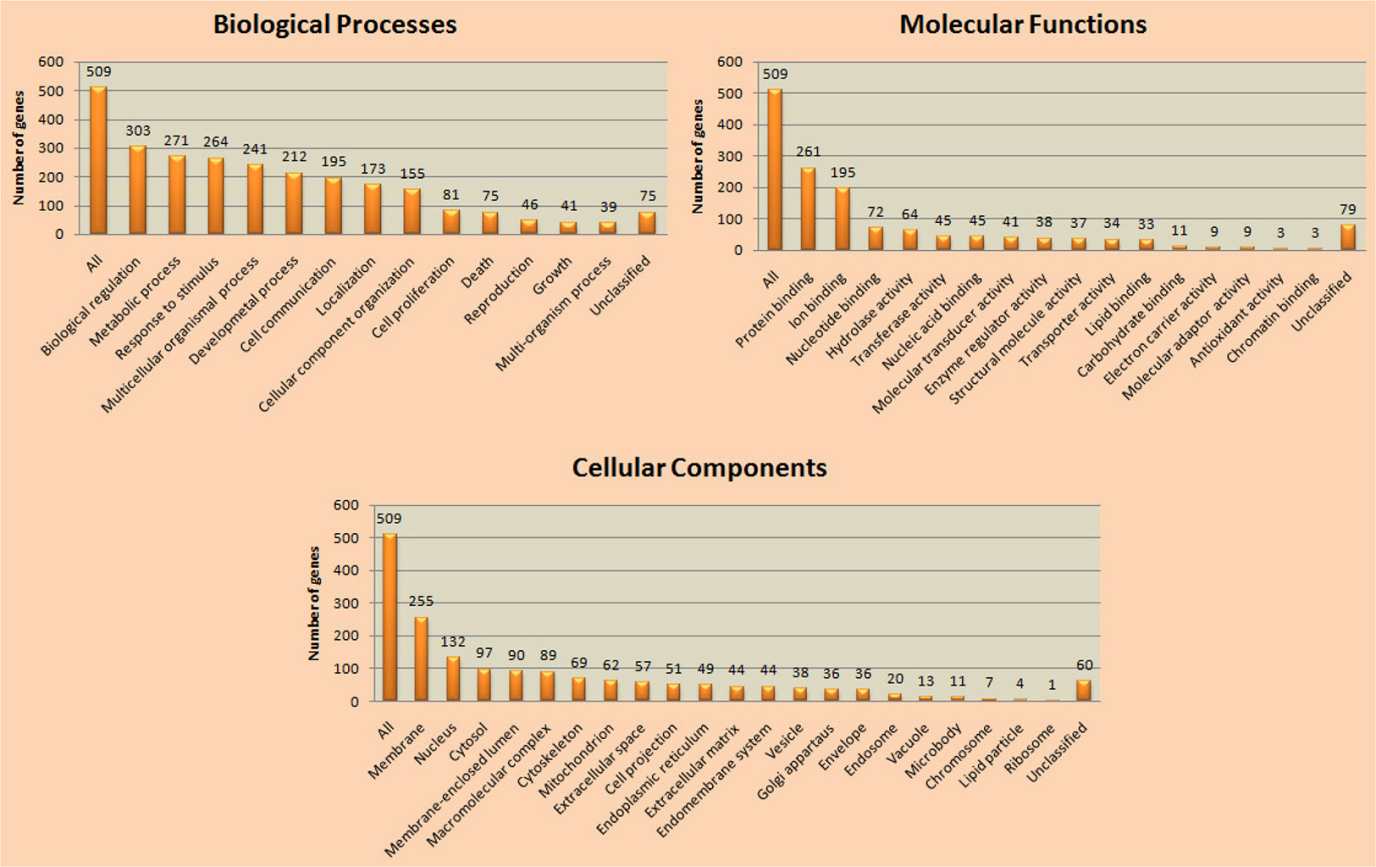
Functional enrichment and annotation analyses. The 631 differentially expressed genes were subjected to manual curation and annotation analyses for their involvement in diverse biological pathways, molecular functions and cellular components.

The majority of identified TFs belonged to zinc-coordinating class and hormone-nuclear receptor family of transcriptional regulatory system. Hepatocyte nuclear factor 4 (*hnf4*), nuclear receptor subfamily 2 group F member 1 (*nr2f1*) and down-regulator of transcription 1 (*dr1*) are the most recurrent TFs regulating genes in early CRC dataset and are the members of same class as well as family of TFs. All these TFs either bind directly or in the form of a complex to control the rate of transcription. This kind of information is primarily required to understand the gene regulation in a comprehensive manner. It is anticipated that for the regulation of genes involved in CRC, manipulation of regulatory region of genes specifically for the identified TFs such as *hnf4*, *nr2f1*, *dr1* and their classes could provide biological insight to experimental biologists and geneticists. Further, an attempt was made to manually curate and annotate the genes for their biological roles, functions, cellular components and their implication in diverse complex biological pathways. Out of 631 differentially expressed genes, functional enrichment for 509 genes was aggravated. Maximum genes had their roles in biological regulation, protein binding and were present at membranes of the cell (Fig 4). This particular section of the manuscript provides an insight to diverse mechanisms and pathways elucidated by the regulation of genes involved in CRC pathway.

**Fig 5:**
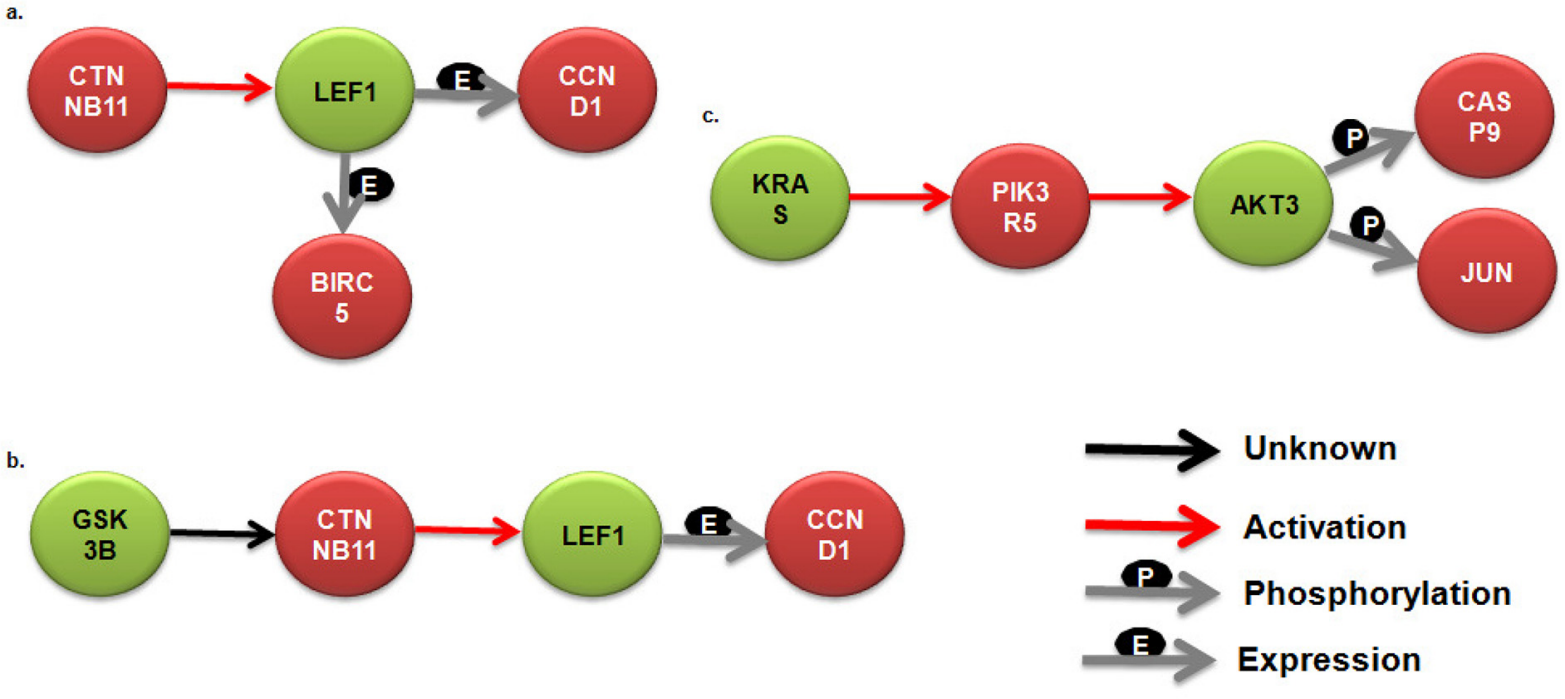
Identified network motifs from colorectal cancer pathway. Some 4 and 5 node sub-graphs have been symbolized with gene names and their interactions if any. If the given interaction in the pathway was found to be missing, it is depicted as unknown (black coloured arrow).

**Fig 6:**
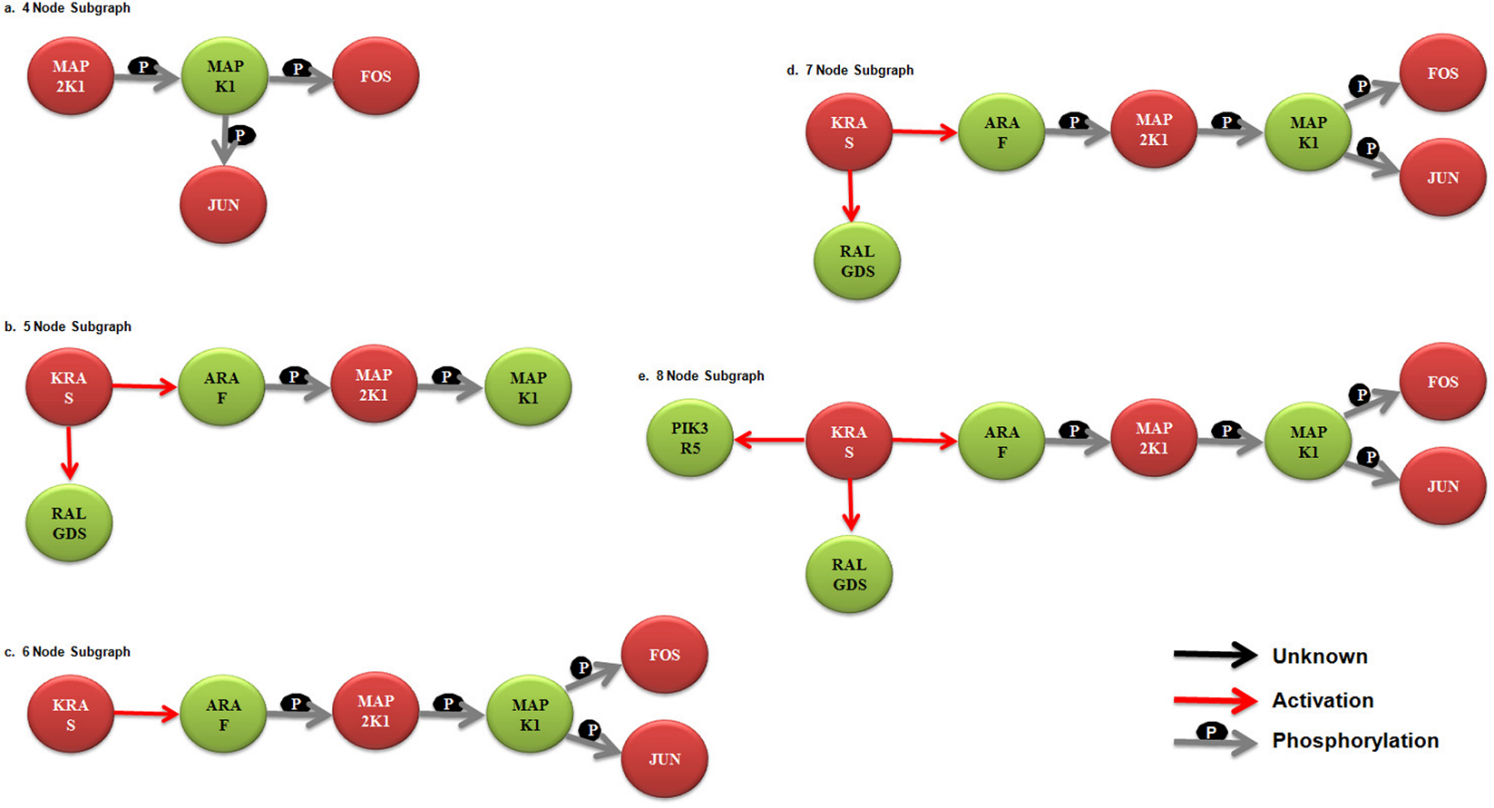
Bottom-up approach for classifying the network motifs. From the 4 to 8 node sub-graphs, each node has been recognized and annotated in order to deduce certain vital interactions.

After acquiring the differential expression pattern, we intended to identify chief sub-networks configured by these genes; facilitating annotation of intricate biological network implicated in CRC. Based on the rationale, detection of crucial network motifs and network patterns was made; providing essential clues concerning the hierarchical decomposition of CRC network. Here the patterns being referred are small connected sub-networks occurring in significantly higher frequencies in a network than would be expected for a given random network. These patterns or motifs are considerably overrepresented and characterize certain essential functional aspects associated with CRC related pathways and its progression. Several motifs ranging from 4–8 sub-graph nodes were generated and annotated for the CRC pathway which is available as supplementary data (available at: http://www.bioinfoindia.org/CRCData), and a few have been depicted in Fig 5. The applied bottom-up approach is clearly demonstrated in Fig 6 starting from 4-node sub-graphs and then proceeding one by one till 8-node sub-graphs were generated; all the interacting genes were annotated along with their functional relationships.

The network motifs thus obtained from CRC pathway contained 4-chain motifs, single input module (SIM), multiple input module (MIM), bifan motifs and other important biological signatures that were supported by significant *z*-scores and *p*-values for their statistical relevance. These network motifs were further subjected to annotation and disease-specific analyses since, they have important functions to execute; as in case of SIM motif, several genes are controlled by a single master gene and the master gene is known to be autoregulatory. Whereas, in MIM motif (a generalization of SIM), a single gene is being controlled by multiple genes [22]. Other regular 4-node motifs confirmed the presence of diamond, biparallel and bifan motifs (often built by two regulatory and two regulated genes). Further, these nodes were annotated for identifying genes involved in these patterns for their biological significance using in house Perl scripts. Similar type of motif graphs were generated for sub-networks of other network sizes and annotation of these graphs were based on statistical criterion via mean-frequencies, standard deviation, *z*-scores and *p*-values.

**Fig 7:**
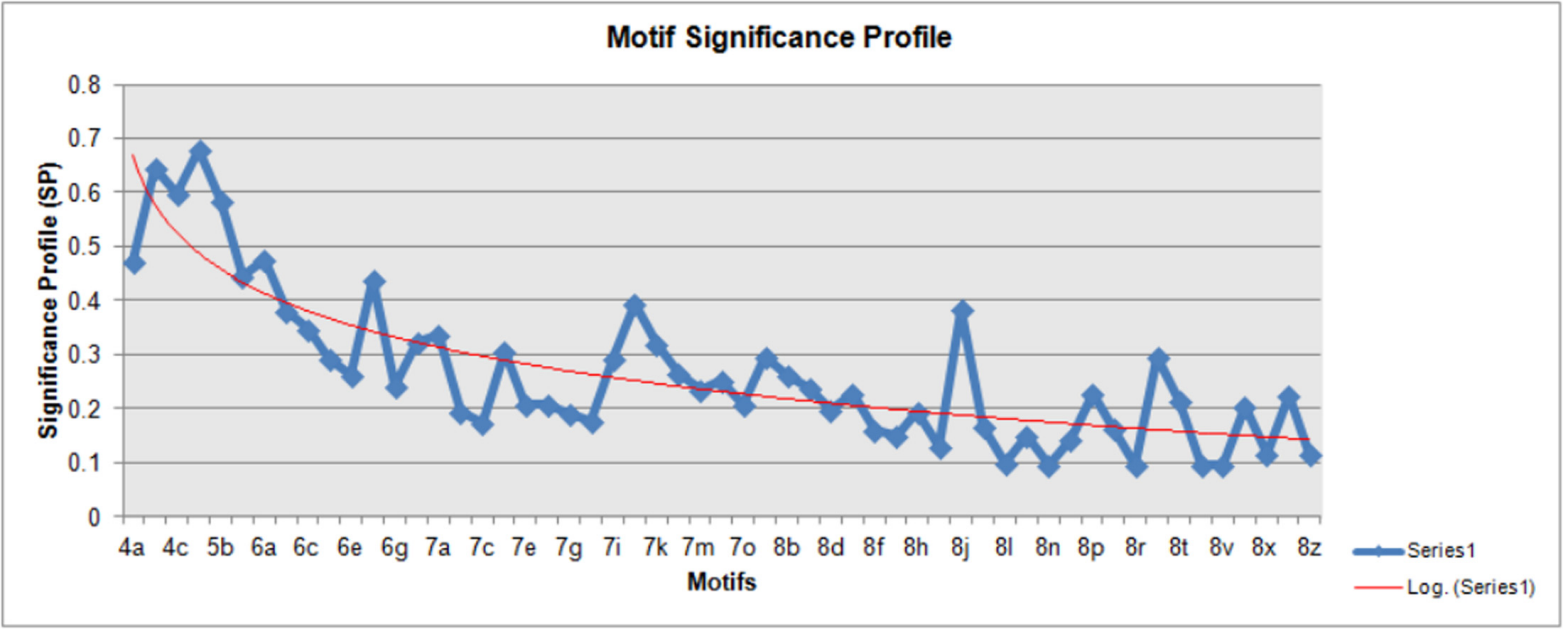
Significance profile for all 4–8 node generated sub-graphs based on normalized *z*-scores. The motif significance profile evidently exemplifies that when the complexity in CRC pathway increases, the interactions among the nodes and intricacy in recognition of genes amplifies immensely. Lesser the node size, it becomes easy to annotate the nodes (genes) and their associations with stronger statistical significance (greater normalized *z*-scores).

The calculated SP was then superlatively plotted on a graph against the different motifs as illustrated in Fig 7. The motif SP graph clearly depicts that as the number of nodes in a motif increase, the complexity increases and further the trend declines representing smaller normalized *z*-score values towards large motif sizes. Based upon this SP profile analysis we suggest that network motifs with smaller node size (3 or 4) are more functionally allied towards their role in pathways while motifs of larger size (> = 5 nodes) are less functional (Fig 7). It is believed that the observed trend might be similar in many such biological networks if analyzed.

The novel deliberated parameters revealed that the lower *‘FT*_*i*_ *’* value proves to be more statistically significant. As it signifies greater involvement of a few genes that explains complex interactions among different nodes in a given motif. Further, the motif showing least *‘FT*_*i*_ *’* value i.e. 0.171 for motif ID ‘7n’ was chosen for identifying key players in the given motif. This information was attained by mapping all genes from the complex CRC pathway onto the network motifs and then frequency of each gene for each network motif was calculated (see S6 Table). This analysis was performed to understand the involvement of different genes on the basis of their occurrence (frequency) in each motif. For instance, consider 4a motif in S6 Table (detail for motif images at http://www.bioinfoindia.org/CRCData), the involvement of *pik3r5*, *kras* and *araf* genes were found4, 5 and 4 times in the same pattern (motif). Finally, a sum of all these frequencies for each gene was calculated to comprehend a cumulative impact and in parallel the frequencies for all genes in the above mentioned motif (with least *‘FT*_*i*_ *’* value) were calculated and presented in Table 3. In general when this approach was applied for 13 DNA repair associated diseases, the least *FTi* value was usually reported for smaller motifs having high SP scores (results unpublished) with exception to results in CRC dataset where least *FTi* value is observed in 7-node motif (i.e. 7n). Therefore, our approach of reducing the entire CRC pathway complexity into smaller sub-graphs and subsequently identifying key players is quite promising as confirmed from Fig 7.

**Table 3:** Putative over-represented genes from CRC pathway as indicated by the most recurrent network motif.

**S. No.**	Genes	Gene Details	Gene Size	Gene Frequency	Molecular Functions	Pubmed IDs_1_
1	KRAS	Kirsten rat sarcoma viral oncogene homolog	21656 Da, 189 amino acids	10	GTPase activity, LRR domain binding, protein binding	19515263, 15069679, 10545700, 19832985
2	ARAF	V-raf murine sarcoma 3611 viral oncogene homolog	67585 Da, 606 amino acids	10	Protein kinase activity, protein binding, ATP binding, transferase activity, metal ion binding	**20145135**
3	PIK3R5	Phosphoinositide-3-kinase, regulatory subunit 5	97348 Da, 880 amino acids	10	G-protein beta/gamma-subunit complex binding, 1-phosphatidylinositol-3-kinase regulator activity	-
4	RALGDS	Ral guanine nucleotide dissociation stimulator	100607 Da, 914 amino acids	10	small GTPase regulator activity, protein binding, guanyl-nucleotide exchange factor activity	**15766656, 17568777**
5	AKT3	V-akt murine thymoma viral oncogene homolog 3	55775 Da, 479 amino acids	8	protein kinase activity, ATP binding, protein binding, transferase activity	18813315
6	RHOA	Ras homolog family member A	21768 Da, 193 amino acids	6	GTPase activity, protein binding, myosin binding, protein domain specific binding	19374769, 11844789, 11953197, 19499974
7	MAP2K1	Mitogen-activated protein kinase kinase 1	43439 Da, 393 amino acids	6	protein kinase activity, ATP binding, protein binding, transferase activity, RAS GTPase binding	**17667937**
8	MAPK1	Mitogen-activated protein kinase 1	41390 Da, 360 amino acids	2	phosphotyrosine binding, DNA binding, protein kinase activity, transferase activity, ATP binding, transcription factor binding	9690379, 11992399
9	GSK3B	Glycogen synthase kinase 3 beta	46744 Da, 420 amino acids	2	protein kinase activity, beta-catenin binding, tau protein binding, transferase activity, p53 binding, NF-kappaB binding	**17640304**
10	BAD	BCL2-associated agonist of cell death	18392 Da, 168 amino acids	2	protein binding, phospholipid binding, protein heterodimerization activity, protein kinase binding, protein phosphatase binding	17583570, 17393317
11	CASP9	Caspase 9, apoptosis-related cysteine peptidase	46281 Da, 416 amino acids	2	cysteine-type endopeptidase activity, enzyme activator activity, protein binding, peptidase activity, SH3 domain binding, protein kinase binding	11912124, 23303631
12	MAPK8	Mitogen-activated protein kinase 8	48296 Da, 427 amino acids	2	catalytic activity, JUN kinase activity, MAP kinase activity, protein kinase activity, ATP binding, phosphotransferase activity,transferase activity, histone deacetylase binding	**19352384, 12819185**

^1^Pubmed IDs correspond to the published literature illustrating role of these genes in colorectal cancer, whereas for some genes, experimental evidences were not found and a few depicted in bold explains their occurrence in colon cancer and further their role in colorectal cancer may be confirmed.

## Discussion

Analyzing complex biological pathway of CRC is a convoluted process and requires an integrative approach for identifying biomarkers for the disease. Thus, the approach we applied not only performs enrichment analyses but also presents observations from many different methods, applications and tools existing for gene expression and network data analyses. The current study intended for identification of vital components in pursuit of reducing the complexity hidden in intricate CRC pathway and their associated biological processes. Identification of crucial network motifs will help systems biologists to find key components from whole pathways and analyze their behaviour against different experimental conditions. Although genes involved in MMR system like *mlh1*, *msh2*, *msh6*, *pms2* and other genes such as *apc* and *mutyh* have already shown their influence on CRC but still cause and progression of the disease remains unrequited. Consequently, we made an effort to identify certain other genes that may potentially impact meticulous understanding of CRC. Many important genes as revealed in Table 3 like kirsten rat sarcoma viral oncogene homolog (*kras*), v-raf murine sarcoma 3611 viral oncogene homolog (*araf*), phosphoinositide-3-kinase, regulatory subunit 5 (*pik3r5*), ral guanine nucleotide dissociation stimulator (*ralgds*) and v-akt murine thymoma viral oncogene homolog 3 (*akt3*) were observed to contribute maximum complexity in the CRC pathway. These genes illustrate higher frequencies and numerous interactions among nodes and are proposed to be vital for CRC disease progression. Here, the CRC pathway complexity has been reduced to a few key genes that may be explored further for their putative roles in the disease.

Previous reports suggest that the mutational analyses of *kras* and *braf* are highly correlated with the development of colorectal cancer by activating MAP kinase pathway [53]. The *braf* gene, an isoform of *araf* (suggested from the pathway level analysis) also has its influence on a number of tumors especially in colorectal and gastric cancer whereas role of *araf* still remains a mystery [54]. Although there have been contradictory reports earlier [55] stating that mutations in *araf* gene may not be associated with pathogenesis of various human cancers. But we found 97% similarity among the two protein sequences (*araf* and *braf*) and the two isoforms share several domains such as Raf_RBD, Pkinase, SPS1, TyrKc and biological properties including binding sites; so intending *araf* as one of the key genes in CRC for its association in disease may prove vital for understanding cancer genetics.

FBJ murine osteosarcoma viral oncogene homolog (*fos*) and jun proto-oncogene (*jun*) with ample frequencies were identified in network motifs as well as in the differential expression dataset depicting their putative roles in forming the convoluted CRC pathway (Figs 5 and 6). As deciphered in the Figures, these genes demonstrate vital interactions among themselves and other genes focussing on activating certain genes, phosphorylating and affecting expression of genes. This study reveals some important markers and a few novel genes and its variants that are believed to associate with CRC and its progression. The 5 genes reported in the study namely, *kras*, *araf*, *pik3r5*, *ralgds* and *akt3* along with 2 other genes *jun* and *fos* can be studied broadly for its association in CRC since, the former genes illustrated complex associations and latter signified high differential expression in diseased state. Moreover, the anticipated genes, *jun*, *fos*, *mapk1*and their REs *znf219*, *hnf4*, *pparg* and *dr1*could be utilized further to control the transcriptional regulation and other regulatory actions executed by these genes. All major responsible candidates were subjected to functional enrichment for their classification in biological processes, pathways and molecular functions they perform. The earlier studies were based on the differential gene expression obtained in early colorectal cancer dataset whereas our approach not only signifies the importance of differentially expressed genes but also helps understand the interactions among these genes/proteins at pathway level. The previous approach revealed seven genes, *cyr61*, *uchl1*, *fos*, *fosb*, *egr1*, *vip*, and *krt24* which were significantly over expressed in diseased as compared to normal. In our study, we propose 5 additional genes *kras*, *araf*, *pik3r5*, *ralgds* and *akt3* along with *jun* and *fos* (also stated by earlier study) which could be explored further for their role in CRC progression.

## Conclusion

The study proposes novel parameters which depicts the dependence of an entire system on a few key genes, proteins and metabolites for examining the statistical significance. Hence, the 5 genes proposed from comprehensive theoretical and computational analysis implicated in CRC may serve as imperative therapeutic targets for CRC. Proposed set of putative TFs will also assist experimental biologists and geneticists to manipulate regulatory processes associated with the genes. There is an imperative need to apply this approach on other perilous diseases as well to identify crucial network components and biomarkers. It is believed that besides key genes proposed in this study, we provide novel methodology to analyze small components of large and complex biological networks. The identified genes from early progression dataset and network analyses for CRC may be explored further and experimentally tested to reveal crucial insights in understanding the disease in an extensive mode.

## Supporting Information

S1 Fig The Self Organizing Map for differentially expressed dataset in colorectal cancer. The darker shades of orange explain clusters having similar expression profiles which then vary to yellow and white for clusters having larger deviations among them.
(TIF)

S2 Fig Sample representations through PCA for control and diseases states for the.cel files of experiments. Clusters represent various conditions. Principal components 1 and 2 are being related through dimensions 1 and 2 respectively.
(TIF)

S3 Fig Sample representations through PCA for control and diseases states for the.cel files of experiments. Clusters represent various conditions. Principal components 1 and 3 are being related through dimensions 1 and 3 respectively.
(TIF)

S1 Table Statistical parameters like *z*-score, *p*-value and significance profile for each network motif. (XLSX)

S2 Table The differentially expressed genes obtained from the CRC microarray dataset. (XLSX)

S3 Table A list of all identified TFs in early CRC progression with their frequency of occurrence. (XLSX)

S4 Table The number of TFs in a particular gene with their relative positions. (XLSX)

S5 Table General description regarding TFs including their class and families. (XLSX)

S6 Table Information on genes with complex interactions occurring in the CRC pathway. (XLSX)

## References

[1] Ferlay J, Soerjomataram I, Ervik M, Dikshit R, Eser S, Mathers C, et al GLOBOCAN 2012 v1.0, Cancer Incidence and Mortality Worldwide: IARC CancerBase No. 11. Lyon, France: International Agency for Research on Cancer; 2013 Available: http://globocan.iarc.fr.

[2] Center MM, Jemal A, Smith RA, Ward E. Worldwide variations in colorectal cancer. CA Cancer J Clin. 2009;59(6):366–78. 10.3322/caac.20038 19897840

[3] Jemal A, Bray F, Center MM, Ferlay J, Ward E, Forman D. Global cancer statistics. CA Cancer J Clin. 2011;61(2):69–90. 10.3322/caac.20107 21296855

[4] Cunningham D, Atkin W, Lenz HJ, Lynch HT, Minsky B, Nordlinger B, et al Colorectal cancer. The Lancet. 2010;375(9719):1030–47. 10.1016/S0140-6736(10)60353-4 20304247

[5] American Cancer Society. Cancer Facts & Figures (2013) Atlanta: American Cancer Society 2013. Available: http://www.cancer.org/research/cancerfactsfigures/cancerfactsfigures/cancer-facts-figures-2013.

[6] Watson AJM, Collins PD. Colon cancer: a civilization disorder. Dig Dis. 2011;29(2):222–8. 10.1159/000323926 21734388

[7] Ferrari P, Jenab M, Norat T, Moskal A, Slimani N, Olsen A, et al Lifetime and baseline alcohol intake and risk of colon and rectal cancers in the European prospective investigation into cancer and nutrition (EPIC). Int J Cancer. 2007;121(9):2065–72. 17640039 10.1002/ijc.22966

[8] Jawad N, Direkze N, Leedham SJ. Inflammatory bowel disease and colon cancer. Recent Results Cancer Res. 2011;185:99–115. 10.1007/978-3-642-03503-6_6 21822822

[9] Astin M, Griffin T, Neal RD, Rose P, Hamilton W. The diagnostic value of symptoms for colorectal cancer in primary care: a systematic review. Br J Gen Pract. 2011;61(586):231–43. 10.3399/bjgp11X572427 PMC3080228 21619747

[10] Edwards BK, Ward E, Kohler BA, Eheman C, Zauber AG, Anderson RN, et al Annual report to the nation on the status of cancer, 1975–2006, featuring colorectal cancer trends and impact of interventions (risk factors, screening, and treatment) to reduce future rates. Cancer. 2010;116(3):544–73. 10.1002/cncr.24760 19998273 PMC3619726

[11] Boyle P, Langman JS. ABC of colorectal cancer: Epidemiology. BMJ: British Medical Journal. 2000;321(7264):805 11009523 10.1136/bmj.321.7264.805 PMC1118620

[12] Levin B, Lieberman DA, McFarland B, Andrews KS, Brooks D, Bond J, et al Screening and surveillance for the early detection of colorectal cancer and adenomatous polyps, 2008: a joint guideline from the American Cancer Society, the US Multi-Society Task Force on Colorectal Cancer, and the American College of Radiology. Gastroenterology. 2008;134(5):1570–95. 10.1053/j.gastro.2008.02.002 18384785

[13] Burt RW, Barthel JS, Dunn KB, David DS, Drelichman E, Ford JM, et al NCCN clinical practice guidelines in oncology. Colorectal cancer screening. J Natl Compr Canc Netw. 2010;8(1):8–61. 20064289 10.6004/jnccn.2010.0003

[14] Whitlock EP, Lin JS, Liles E, Beil TL, Fu R. Screening for Colorectal Cancer: A Targeted, Updated Systematic Review for the U.S. Preventive Services Task Force. Annals of Internal Medicine. 2008;149(9):638–58. 18838718 10.7326/0003-4819-149-9-200811040-00245

[15] Pulido EG, Oliveira AR, Bargues JB, Ponce CG, Carrato A. Molecular biology of colorectal cancer In: Cidon EU, editor. The Challenge of Colorectal Cancer: A Review Book. India: Research Signpost; 2011.

[16] Pino MS, Chung DC. The chromosomal instability pathway in colon cancer. Gastroenterology. 2010;138(6):2059–72. 10.1053/j.gastro.2009.12.065 20420946 PMC4243705

[17] Boland CR, Goel A. Microsatellite Instability in Colorectal Cancer. Gastroenterology. 2010;138(6):2073–87.e3. 10.1053/j.gastro.2009.12.064 20420947 PMC3037515

[18] Sinicrope FA, Sargent DJ. Molecular pathways: microsatellite instability in colorectal cancer: prognostic, predictive, and therapeutic implications. Clin Cancer Res. 2012;18(6):1506–12. 10.1158/1078-0432.CCR-11-1469 22302899 PMC3306518

[19] Armaghany T, Wilson JD, Chu Q, Mills G. Genetic alterations in colorectal cancer. Gastrointest Cancer Res. 2012;5(1):19–27. 22574233 PMC3348713

[20] Wheeler JM, Bodmer WF, Mortensen NJ. DNA mismatch repair genes and colorectal cancer. Gut. 2000;47(1):148–53. 10861278 10.1136/gut.47.1.148 PMC1727951

[21] Sehgal M, Singh TR. DR-GAS: a database of functional genetic variants and their phosphorylation states in human DNA repair systems. DNA Repair (Amst). 2014;16:97–9103. 24548788 10.1016/j.dnarep.2014.01.004

[22] Panigrahi PP, Singh TR. Computational studies on Alzheimer's disease associated pathways and regulatory patterns using microarray gene expression and network data: revealed association with aging and other diseases. J Theor Biol. 2013;334:109–21. 10.1016/j.jtbi.2013.06.013 23811083

[23] Tan WY, Yan XW. A new stochastic and state space model of human colon cancer incorporating multiple pathways. Biol Direct. 2010;5:26 10.1186/1745-6150-5-26 20406446 PMC2875223

[24] Hegde P, Qi R, Gaspard R, Abernathy K, Dharap S, Earle HJ, et al Identification of Tumor Markers in Models of Human Colorectal Cancer Using a 19,200-Element Complementary DNA Microarray. Cancer Research. 2001;61(21):7792–7. 11691794

[25] Zou T-T, Selaru FM, Xu Y, Shustova V, Yin J, Mori Y, et al Application of cDNA microarrays to generate a molecular taxonomy capable of distinguishing between colon cancer and normal colon. Oncogene. 2002;21(31):4855–62. 12101425 10.1038/sj.onc.1205613

[26] The Gene Ontology C, Ashburner M, Ball CA, Blake JA, Botstein D, Butler H, et al Gene Ontology: tool for the unification of biology. Nature genetics. 2000;25(1):25–9. 10802651 10.1038/75556 PMC3037419

[27] Pratap A, Taliyan S, Singh TR. NMDB: Network Motif Database envisaged and explicated from human disease specific pathways. Journal of biological systems. 2014;22:89–100.

[28] Barrett T, Wilhite SE, Ledoux P, Evangelista C, Kim IF, Tomashevsky M, et al NCBI GEO: archive for functional genomics data sets-update. Nucleic Acids Research. 2013;41(Database issue):D991–D5. 10.1093/nar/gks1193 23193258 PMC3531084

[29] Hong Y, Ho KS, Eu KW, Cheah PY. A susceptibility gene set for early onset colorectal cancer that integrates diverse signaling pathways: implication for tumorigenesis. Clin Cancer Res. 2007;13(4):1107–14. 17317818 10.1158/1078-0432.CCR-06-1633

[30] Baglioni S, Genuardi M. Simple and complex genetics of colorectal cancer susceptibility. American Journal of Medical Genetics Part C: Seminars in Medical Genetics. 2004;129C(1):35–43. 10.1002/ajmg.c.30023 15264271

[31] Lynch HT, de la Chapelle A. Hereditary colorectal cancer. N Engl J Med. 2003;348(10):919–32. 12621137 10.1056/NEJMra012242

[32] Sehgal M, Singh TR. Identification and analysis of biomarkers for mismatch repair proteins: A bioinformatic approach. J Nat Sci Biol Med. 2012;3(2):139–46. 10.4103/0976-9668.101887 23225975 PMC3510907

[33] Kanehisa M, Goto S. KEGG: Kyoto Encyclopedia of Genes and Genomes. Nucleic Acids Research. 2000;28(1):27–30. 10592173 10.1093/nar/28.1.27 PMC102409

[34] Joshi-Tope G, Vastrik I, Gopinath GR, Matthews L, Schmidt E, Gillespie M, et al The Genome Knowledgebase: a resource for biologists and bioinformaticists. Cold Spring Harb Symp Quant Biol. 2003;68:237–43. 15338623 10.1101/sqb.2003.68.237

[35] Stark C, Breitkreutz BJ, Reguly T, Boucher L, Breitkreutz A, Tyers M. BioGRID: a general repository for interaction datasets. Nucleic Acids Research. 2006;34(Database issue):D535–D9. 16381927 10.1093/nar/gkj109 PMC1347471

[36] Kandasamy K, Mohan SS, Raju R, Keerthikumar S, Kumar GSS, Venugopal AK, et al NetPath: a public resource of curated signal transduction pathways. Genome Biol. 2010;11(1):R3 10.1186/gb-2010-11-1-r3 20067622 PMC2847715

[37] Irizarry RA, Hobbs B, Collin F, Beazer-Barclay YD, Antonellis KJ, Scherf U, et al Exploration, normalization, and summaries of high density oligonucleotide array probe level data. Biostatistics. 2003;4(2):249–64. 12925520 10.1093/biostatistics/4.2.249

[38] Bender R, Lange S. Adjusting for multiple testing—when and how? J Clin Epidemiol. 2001;54(4):343–9. 11297884 10.1016/s0895-4356(00)00314-0

[39] Zang S, Guo R, Zhang L, Lu Y. Integration of statistical inference methods and a novel control measure to improve sensitivity and specificity of data analysis in expression profiling studies. J Biomed Inform. 2007;40(5):552–60. 17317331 10.1016/j.jbi.2007.01.002

[40] Tusher VG, Tibshirani R, Chu G. Significance analysis of microarrays applied to the ionizing radiation response. Proc Natl Acad Sci U S A. 2001;98(9):5116–21. 11309499 10.1073/pnas.091062498 PMC33173

[41] Covell DG, Wallqvist A, Rabow AA, Thanki N. Molecular classification of cancer: unsupervised self-organizing map analysis of gene expression microarray data. Mol Cancer Ther. 2003;2(3):317–32. 12657727

[42] Hotelling H. Analysis of a complex of statistical variables into principle components. J Educ Psychol. 1933;24:417–41.

[43] Gotea V, Ovcharenko I. DiRE: identifying distant regulatory elements of co-expressed genes. Nucleic Acids Research. 2008;36(Web Server issue):W133–W9. 10.1093/nar/gkn300 18487623 PMC2447744

[44] Ho Sui SJ, Fulton DL, Arenillas DJ, Kwon AT, Wasserman WW. oPOSSUM: integrated tools for analysis of regulatory motif over-representation. Nucleic Acids Res. 2007;35(Web Server issue):245–52. 10.1093/nar/gkm427 PMC1933229 17576675

[45] Zhang B, Kirov S, Snoddy J. WebGestalt: an integrated system for exploring gene sets in various biological contexts. Nucleic Acids Research. 2005;33(Web Server issue):W741–W8. 15980575 10.1093/nar/gki475 PMC1160236

[46] Eden E, Navon R, Steinfeld I, Lipson D, Yakhini Z. GOrilla: a tool for discovery and visualization of enriched GO terms in ranked gene lists. BMC Bioinformatics. 2009;10:48 10.1186/1471-2105-10-48 19192299 PMC2644678

[47] Eden E, Lipson D, Yogev S, Yakhini Z. Discovering motifs in ranked lists of DNA sequences. PLoS Comput Biol. 2007;3(3):e39 17381235 10.1371/journal.pcbi.0030039 PMC1829477

[48] Kashtan N, Itzkovitz S, Milo R, Alon U. Efficient sampling algorithm for estimating subgraph concentrations and detecting network motifs. Bioinformatics. 2004;20(11):1746–58. 15001476 10.1093/bioinformatics/bth163

[49] Schreiber F, Schwöbbermeyer H. MAVisto: a tool for the exploration of network motifs. Bioinformatics. 2005;21(17):3572–4. 16020473 10.1093/bioinformatics/bti556

[50] Wernicke S, Rasche F. FANMOD: a tool for fast network motif detection. Bioinformatics. 2006;22(9):1152–3. 16455747 10.1093/bioinformatics/btl038

[51] Fruchterman TMJ, Reingold EM. Graph drawing by force-directed placement. Software: practice and experience 1991;21:1129–64.

[52] Wernicke S. Efficient detection of network motifs. IEEE/ACM Transactions on Computational Biology and Bioinformatics (TCBB). 2006;3(4):347–59. 10.1109/TCBB.2006.51 17085844

[53] Fransén K, Klintenäs M, Österström A, Dimberg J, Monstein HJ, Söderkvist P. Mutation analysis of the BRAF, ARAF and RAF-1 genes in human colorectal adenocarcinomas. Carcinogenesis. 2004;25(4):527–33. 14688025 10.1093/carcin/bgh049

[54] Matallanas D, Birtwistle M, Romano D, Zebisch A, Rauch J, von Kriegsheim A, et al Raf family kinases: old dogs have learned new tricks. Genes Cancer. 2011;2(3):232–60. 10.1177/1947601911407323 21779496 PMC3128629

[55] Lee JW, Soung YH, Kim SY, Park WS, Nam SW, Min WS, et al Mutational analysis of the ARAF gene in human cancers. Apmis. 2005;113(1):54–7. 15676015 10.1111/j.1600-0463.2005.apm1130108.x

