# Supplementary figures and images for: An Integrative Approach for Mapping Differentially Expressed Genes and Network Components Using Novel Parameters to Elucidate Key Regulatory Genes in Colorectal Cancer
Source: PLoS One. 2015 Jul 29;10(7):e0133901. doi: 10.1371/journal.pone.0133901 (PMC4519280; doi:10.1371/journal.pone.0133901)

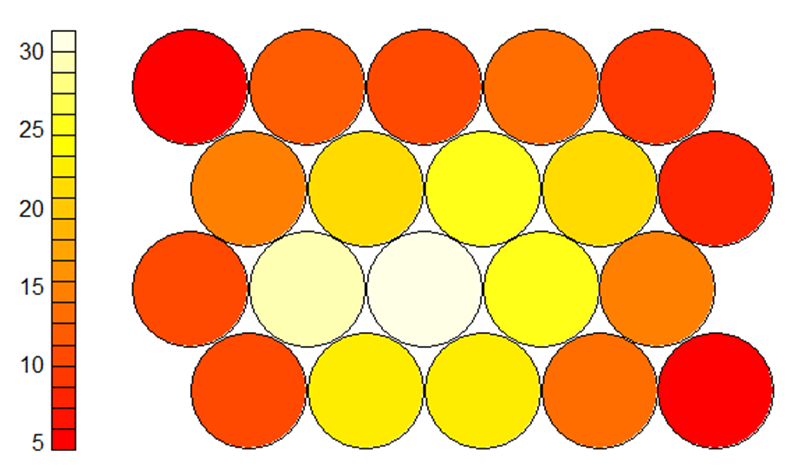

Supplement: S1 Fig — The darker shades of orange explain clusters having similar expression profiles which then vary to yellow and white for clusters having larger deviations among them. (TIF) [file pone.0133901.s001.tif]

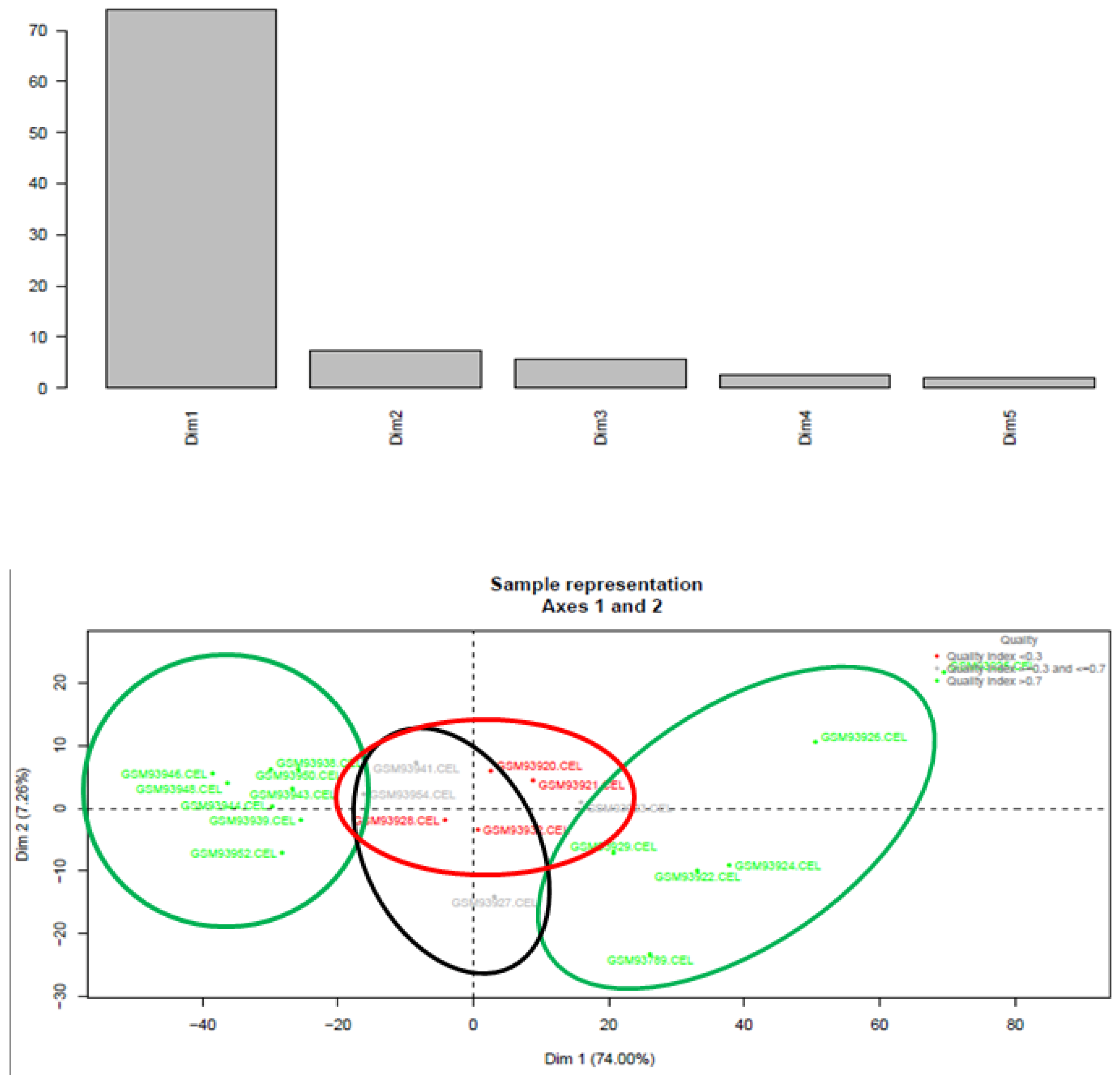

Supplement: S2 Fig — Clusters represent various conditions. Principal components 1 and 2 are being related through dimensions 1 and 2 respectively. (TIF) [file pone.0133901.s002.tif]

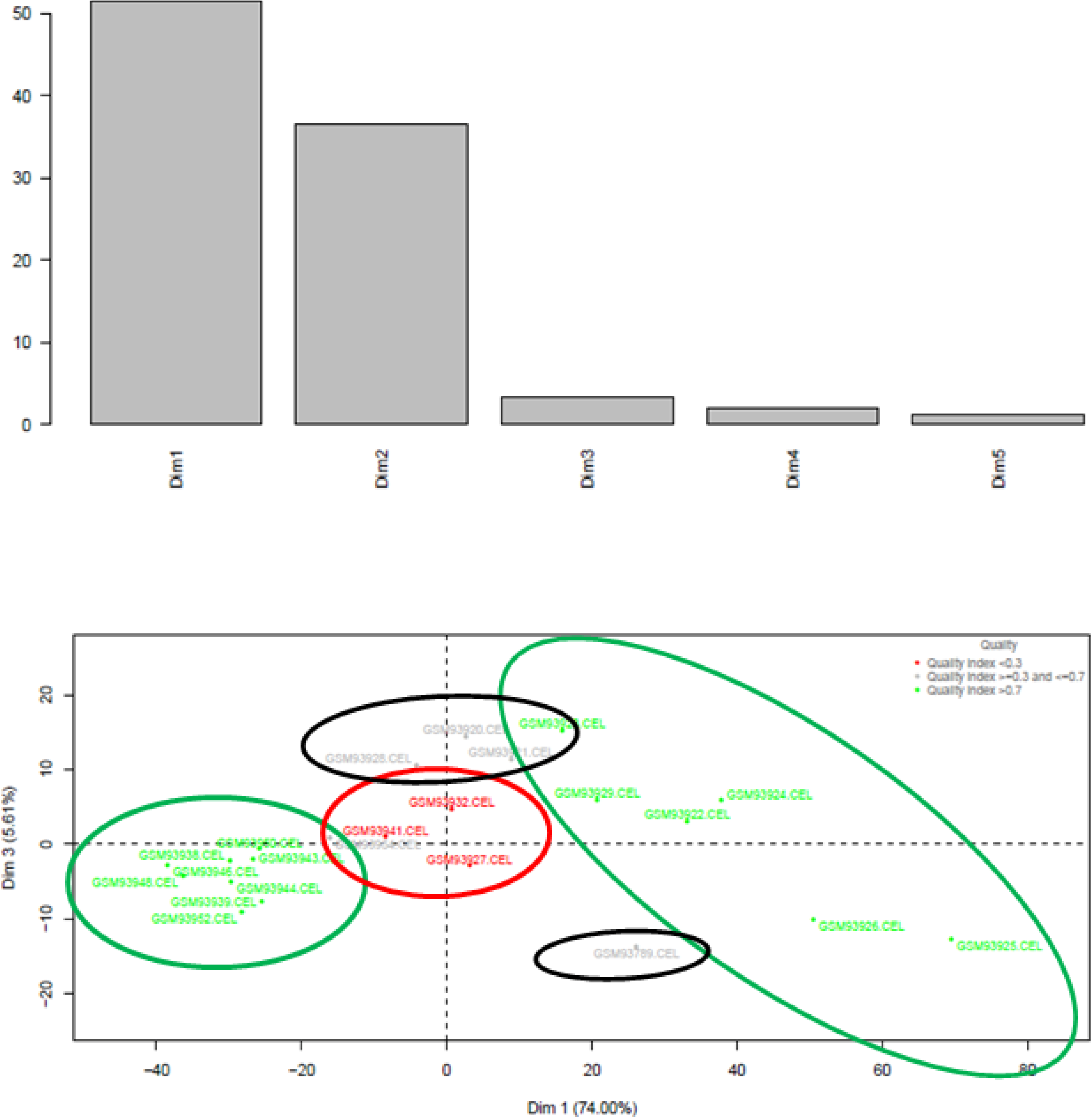

Supplement: S3 Fig — Clusters represent various conditions. Principal components 1 and 3 are being related through dimensions 1 and 3 respectively. (TIF) [file pone.0133901.s003.tif]
